# Supplementary material for: Genetic Diversity of Montenegrin Local Sheep Breeds Based on Microsatellite Markers
Source: Animals (Basel). 2022 Nov 3;12(21):3029. doi: 10.3390/ani12213029 (PMC9653887; doi:10.3390/ani12213029)
Supplement: Supplementary file 1 [file animals-12-03029-s001.zip › animals-1979395-supplementary.pdf]

**Supplemment data:**

**Table S1.** Hardy–Weinberg (HW) equilibrium test in all studied microsatellite loci by breed.

| Locus | JP | SOR | ZZ | BAR | LJAB | SJ | PIP |
|---|---|---|---|---|---|---|---|
| AE129 | 0.221 ns | 0.298 ns | 0.628 ns | 0.536 ns | 0.883 ns | 0.323 ns | 0.864 ns |
| CP49 | 0.997 ns | 0.006 ** | 0.983 ns | 0.250 ns | 0.714 ns | 0.650 ns | 0.746 ns |
| CSRD247 | 0.994 ns | 0.983 ns | 0.605 ns | 0.007 * | 0.646 ns | 0.601 ns | 0.783 ns |
| FCB20 | 0.410 ns | 0.417 na | 0.939 ns | 0.299 ns | 0.326 ns | 0.592 ns | 0.033 * |
| HSC | 0.986 ns | 0.530 ns | 0.995 ns | 0.029 * | 0.742 ns | 0.159 ns | 0.911 ns |
| ILSTS005 | 0.001 *** | 0.814 ns | 0.991 ns | 0.000 *** | 0.855 ns | 0.996 ns | 0.543 ns |
| ILSTS011 | 0.934 ns | 0.434 ns | 0.989 ns | 0.216 ns | 0.634 ns | 0.575 ns | 0.546 ns |
| INRA006 | 0.507 ns | 0.990 ns | 0.998 ns | 0.003 ** | 0.996 ns | 0.708 ns | 0.249 ns |
| INRA023 | 0.803 ns | 0.704 ns | 0.122 ns | 0.333 ns | 0.138 ns | 0.845 ns | 0.876 ns |
| INRA049 | 0.681 ns | 0.062 ns | 0.914 ns | 0.600 ns | 0.227 ns | 0.706 ns | 0.804 ns |
| INRA063 | 0.112 ns | 0.972 ns | 0.898 ns | 0.719 ns | 0.741 ns | 0.002 ** | 0.234 ns |
| INRA132 | 0.110 ns | 0.566 ns | 0.896 ns | 0.000 *** | 0.115 ns | 0.916 ns | 0.456 ns |
| INRA172 | 0.747 ns | 0.470 ns | 0.733 ns | 0.960 ns | 0.037 * | 0.803 ns | 0.923 ns |
| MAF214 | 0.723 ns | 0.802 ns | 0.377 ns | 0.012 * | 0.939 ns | 0.018 ** | 0.443 ns |
| MAF65 | 0.179 ns | 0.292 ns | 0.831 ns | 0.002 ** | 0.942 ns | 0.174 ns | 0.642 ns |
| McM042 | 0.798 ns | 0.972 ns | 0.842 ns | 0.987 ns | 0.211 ns | 0.584 ns | 0.974 ns |
| SPS113 | 0.060 ns | 0.116 ns | 0.811 ns | 0.336 ns | 0.002 ** | 0.970 ns | 0.505 ns |
| SPS115 | 1.000 ns | 0.671 ns | 0.553 ns | 0.000 *** | 0.003 ** | 0.056 ns | 0.119 ns |

Abbreviations: JP—Jezeropivska, SOR—Sora, Zeta zuja—ZZ, Bardoka—BAR, Ljaba—LJAB, Sjenička—SJ, Piperska zuja—PIP. Significant $p$ values: ns = not significant, * $p < 0.05$, ** $p < 0.01$, *** $p < 0.001$.
